# A Comprehensive Evaluation of Lymph Node Staging and a Proposal to Subdivide N2b Category in Colorectal Cancer Patients

**DOI:** 10.3390/cancers17244002

**Published:** 2025-12-16

**Authors:** Kexing Xi, Yunlong Wu, Lin Feng, Yuelu Zhu, Hui Fang, Haizeng Zhang

**Affiliations:** 1 Department of Colorectal Surgery, National Cancer Center/National Clinical Research Center for Cancer/Cancer Hospital, Chinese Academy of Medical Sciences and Peking Union Medical College, Beijing 100021, China; xikxing@mail3.sysu.edu.cn (K.X.); dr_wuyunl@mail.ccmu.edu.cn (Y.W.); 2 State Key Laboratory of Molecular Oncology, National Cancer Center/National Clinical Research Center for Cancer/Cancer Hospital, Chinese Academy of Medical Sciences and Peking Union Medical College, Beijing 100021, China; fenglin@cicams.ac.cn; 3 Department of Pathology, National Cancer Center/National Clinical Research Center for Cancer/Cancer Hospital, Chinese Academy of Medical Sciences and Peking Union Medical College, Beijing 100021, China; zhuyuelu@cicams.ac.cn; 4 Department of Radiotherapy, National Cancer Center/National Clinical Research Center for Cancer/Cancer Hospital, Chinese Academy of Medical Sciences and Peking Union Medical College, Beijing 100021, China; fanghuii@163.com

**Keywords:** colorectal cancer, prognosis, number of positive lymph node, stage N2b

## Abstract

**Objective**: This study aimed to assess the impact of the number of metastatic lymph nodes (LNs) on survival and propose a subdivision of the N2b category in colorectal cancer (CRC) patients. **Methods**: We retrospectively analyzed from two sources: clinicopathologic data of CRC patients with stage pTxN2bM0 who initially underwent radical surgery at Cancer Hospital, Chinese Academy of Medical Sciences/National Cancer center (NCC), and patients with stage pTxN0-2bM0-1 in the Surveillance, Epidemiology and End Results (SEER) database from January 2010 to December 2015. The optimal cutoff value of the number of positive lymph nodes (PLNs) was determined based on the principle of maximum chi-square value. We constructed survival curves using the Kaplan–Meier method, assessed survival differences with the log-rank test, and conducted univariate and multivariate analyses using the Cox proportional hazard regression model. **Results**: A total of 68,335 CRC patients were included: 240 from the NCC cohort, and 68,095 from the SEER cohort. Within the SEER cohort, 65,189 patients had M0 stage disease and 2,906 had M1 stage disease. The optimal PLN cutoff value determined by X-tile software (Version 3.6.1) was 13. According to PLN, stage N2b patients were divided into two groups: stage N2b# (7 ≤ PLN < 13) and stage N3 (PLN ≥ 13). In the NCC cohort, the 5-year overall survival (OS) rates of stage N2b# and N3 patients were 66.0% and 45.7%, respectively (*p* < 0.001). In the SEER cohort, the 5-year cancer-specific survival (CSS) rate was 57.1% for stage N2b# patients compared with 40.2% for stage N3 patients (*p* < 0.001). The results of multivariate Cox analysis demonstrated that modified stage pN was the independent prognosis factor of OS in the NCC cohort (HR = 1.869, 95%CI:1.253–2.787, *p* = 0.002); modified stage pN was also the independent prognosis indicator of CSS in the SEER cohort (N3:N0, HR = 8.170, 95%CI: 7.298–9.146, *p* < 0.001). There was no survival difference between TxN3M0 and TxN0-2b#M0 (5-year CSS rate: 40.2% vs. 30.1%, *p* = 0.050; 5-year OS rate: 35.3% vs. 27.8%, *p* = 0.358). **Conclusions**: The N category served as a strong independent prognostic indicator in CRC patients. Furthermore, PLN emerged as an independent prognostic factor specifically in stage N2b CRC patients. These findings suggest that clinicians may utilize PLN for prognostic stratification and tailor adjuvant therapeutic strategies accordingly for patients diagnosed with stage N2b CRC.

## Simple Summary

This study aimed to assess the impact of the number of metastatic lymph nodes (LNs) on survival and propose a subdivision of the N2b category in colorectal cancer (CRC) patients. We found that the N category served as a strong independent prognostic indicator in CRC patients. Furthermore, the number of positive lymph nodes (PLNs) emerged as an independent prognostic factor specifically in stage N2b CRC patients. Clinicians may utilize PLNs for prognostic stratification and tailor adjuvant therapeutic strategies accordingly for patients diagnosed with stage N2b CRC.

## 1. Introduction

Colorectal cancer (CRC) is one of the most common cancers, and its incidence has been steadily rising over the past 30 years worldwide [1,2]. Currently, Surgery is the primary treatment modality for CRC [3,4]. Metastasis is the main cause of treatment failure and death. The lymph nodes (LNs) are considered the earliest and most frequent sites of metastasis, as well as the most significant prognostic factor in CRC [5]. Consequently, the examination and evaluation of LNs play a pivotal role in predicting the prognosis of CRC patients [6]. Current guidelines, such as those from the American Joint Committee on Cancer (AJCC) and the National Comprehensive Cancer Network (NCCN), advocate the examination of a minimum of 12 LNs for accurate N staging in CRC patients [3,7]. According to the International Union Against Cancer (UICC) and AJCC TNM staging system, the N stage can be categorized as N0 (no LN metastasis), N1 (1–3 LNs with metastasis), or N2 (≥4 LNs with metastasis). Moreover, N2 is further divided into N2a (4–6 LNs with metastasis) and N2b (≥7 LNs with metastasis) subcategories [8].

Numerous studies have highlighted that LN metastasis is a key prognostic factor in colorectal cancer [5,9,10], with poorer survival outcomes following an increased number of positive LNs [11]. Consequently, the prognosis of N2b stage CRC is worst in M0 patients. However, the Current TNM staging system classifies all patients with 7 or more positive LNs into the same N2b stage without considering the impact of varying numbers of positive LNs within this subgroup. Therefore, N2b CRC patients display a wide range in the number of positive LNs and have heterogeneous prognoses. Some N2b CRC patients may even have more than 50 positive LNs, and their prognosis is very poor. Therefore, it might not be appropriate to uniformly classify all CRC patients with ≥7 positive LNs into stage N2b, as this fails to capture the diversity in prognosis within this subgroup. Consequently, it is necessary to further subdivide N2b to offer more precise prognostic information.

In this study, we collected and reviewed the data of CRC patients from the Cancer Hospital, Chinese Academy of Medical Sciences/National Cancer Center (NCC) and the Surveillance, Epidemiology and End Results (SEER) database to explore the relationship between the number of LNs with metastasis and the prognosis of N2b CRC patients. The former and the latter served as the development cohort and validation cohort, respectively. The objective of our study is to subdivide N2b and provide more precise prognostic information to patients.

## 2. Materials and Methods

### 2.1. Study Population

#### 2.1.1. NCC Cohort

This study included CRC patients who underwent surgery at the Cancer Hospital, Chinese Academy of Medical Sciences from January 2010 to December 2015 and were histologically confirmed to be stage pTxN2bM0. The exclusion criteria were as follows: (1) Patients who received neoadjuvant therapy; (2) Patients with hereditary nonpolyposis colon cancer or familial polyposis; (3) Patients who underwent local resection or palliative surgery; (4) Histopathological diagnoses other than adenocarcinoma; (5) Distant metastatic disease found preoperatively or intraoperatively; (6) Incomplete TNM staging information; (7) Patients with zero examined LNs or incomplete LN evaluation data; (8) Patients with a second tumor; (9) Individuals who died within 1 month post-surgery; (10) Patients aged over 85 years or under 18 years; and (11) Patients with Inflammatory Bowel disease. Survival analysis in this cohort was based on overall survival (OS).

#### 2.1.2. SEER Cohort (M0 Cases)

The SEER database was used to identify CRC patients who initially underwent surgery between January 2010 and December 2015 and were confirmed to have stage pTxN0-2bM0 based on pathology. The exclusion criteria for the cohort were identical to those of the NCC cohort.

#### 2.1.3. SEER Cohort (M1 Cases)

For CRC patients in the SEER database who underwent surgery for both primary and metastatic tumors between January 2010 and December 2015 and were confirmed to have stage pTxN0-2bM1, the following exclusion criteria were applied: (1) Patients who received neoadjuvant therapy; (2) Patients diagnosed with hereditary nonpolyposis colon cancer or familial polyposis; (3) Cases diagnosed with stage M1NOS (the site of metastasis was unclear); (4) Histopathological diagnoses other than adenocarcinoma; (5) Incomplete TNM staging information; (6) Cases with zero examined LNs or incomplete LN evaluation data; (7) Patients with a second tumor; (8) Individuals who died within 1 month post-surgery; and (9) Patients aged over 85 years or under 18 years.

Survival analysis of these two SEER cohorts was based on cancer-specific survival (CSS).

### 2.2. Statistical Analysis

Survival analysis was conducted to assess differences in survival rates using the log-rank test. The Kaplan–Meier method was employed to generate the survival curves. We calculated the hazard ratio (HR) and its corresponding 95% confidence interval (CI) for both OS and CSS using univariate and multivariate Cox regression models. The optimal cutoff value for the number of positive lymph nodes (PLNs) was determined using X-tile (Version 3.6.1, Yale University) based on the principle of maximum chi-square value. The other data analysis was performed using SPSS 25.0 software (SPSS Inc., Chicago, IL, USA). A two-sided *p* value < 0.05 was considered statistically significant.

## 3. Results

### 3.1. Clinicopathologic Characteristics of Patients

In the NCC cohort, a total of 240 CRC patients were enrolled. Over half of the patients were male (141/240, 58.8%), and 61.3% (147/240) were under 60 years old. Most of the patients received laparoscopic surgery (153/240, 63.8%). A majority of the patients had more than 12 examined LNs (234/240, 97.5%). The distribution of patients in terms of T stage was as follows: 3.8% (9/240) with T1–2, 61.3% (147/240) with T3, and 35.0% (84/240) with T4. Adjuvant chemotherapy was administered to 85.8% (206/240) of the patients. The clinicopathologic characteristics of these patients are summarized in Table 1.

In the SEER cohort, 65,189 M0 patients and 2906 M1 patients were included in this study. Among the M0 patients, nearly half of patients were male. A total of 25,207 (38.7%) were ≤60 years old, and the majority were of white ethnicity. A total of 56.1% (36,577/65,189) of the patients were married. In terms of differentiation grade, more than half of the patients were moderately differentiated. The distribution of N stages was as follows: 62.9% (41,004/65,189) with N0, 11.8% (7666/65,189) with N1a, 11.5% (7465/65,189) with N1b, 1.6% (1017/65,189) with N1c, 6.9% (4517/65,189) with N2a, and 5.4% (3520/65,189) with N2b. The clinicopathologic characteristics of these patients are shown in Table 2.

### 3.2. Optimal PLN Cutoff Value for Substage N2b Patients

**Figure 1:**
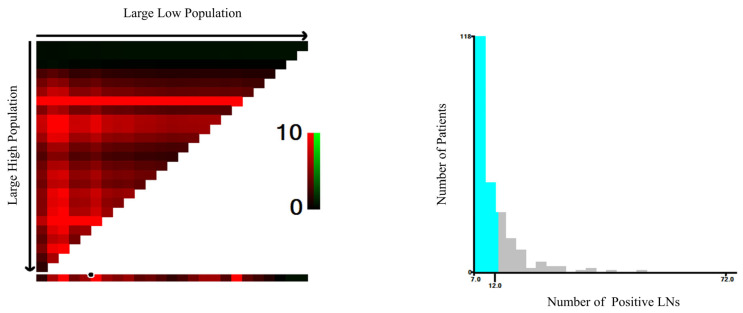
Optimal cut-off value for PLN identified by X-tile analysis. Cyan represents patients with 7 to 12 positive lymph nodes, while gray represents patients with 13 or more positive lymph nodes.

Using data from the NCC cohort, the optimal cutoff value for PLNs was determined to be 13, based on the principle of maximum chi-square value, to best distinguish survival between two newly defined N2b subgroups (Figure 1). According to this result, patients with 7–12 metastatic LNs and those with ≥13 metastatic LNs were defined as stage N2b# and stage N3, respectively.

### 3.3. Survival Analysis in the NCC Cohort

**Figure 2:**
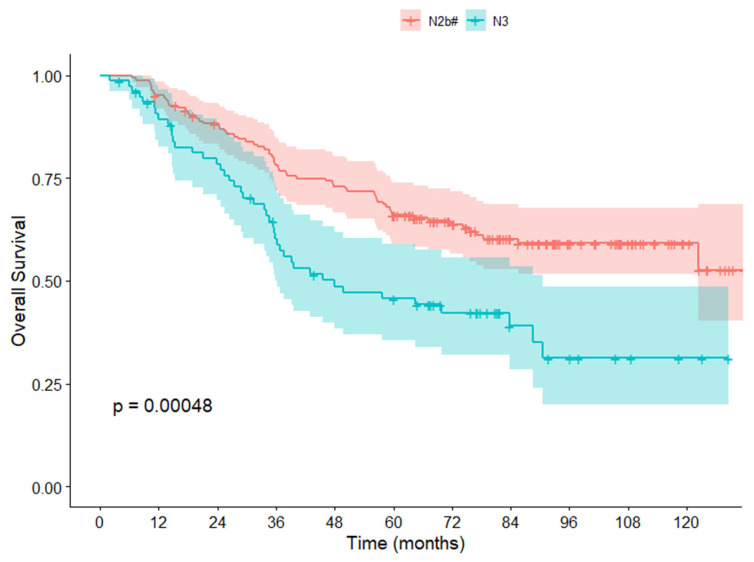
Overall survival curves for TxN2b#M0 and TxN3M0 patients in the NCC cohort.

Using the cutoff value chosen, the N2b patients in the NCC cohort were divided into two groups: stage N2b# (163 cases) and stage N3 (77 cases). The stage N3 patients had worse survival compared to stage N2b# patients, with a 5-year OS rate of 45.7% and 66.0%, respectively (*p* < 0.001) (Figure 2). Univariate analysis identified several factors significantly associated with OS, including age, preoperative CEA, preoperative CA199, tumor location, vascular invasion, perineural invasion, differentiation grade, pT stage, modified pN stage, and adjuvant chemotherapy (*p* < 0.001). Multivariate analysis revealed that age, preoperative CA199, tumor location, perineural invasion, pT stage, modified pN stage, and adjuvant chemotherapy independently influenced OS (Table 1).

### 3.4. Validation in the SEER Cohort

**Figure 3:**
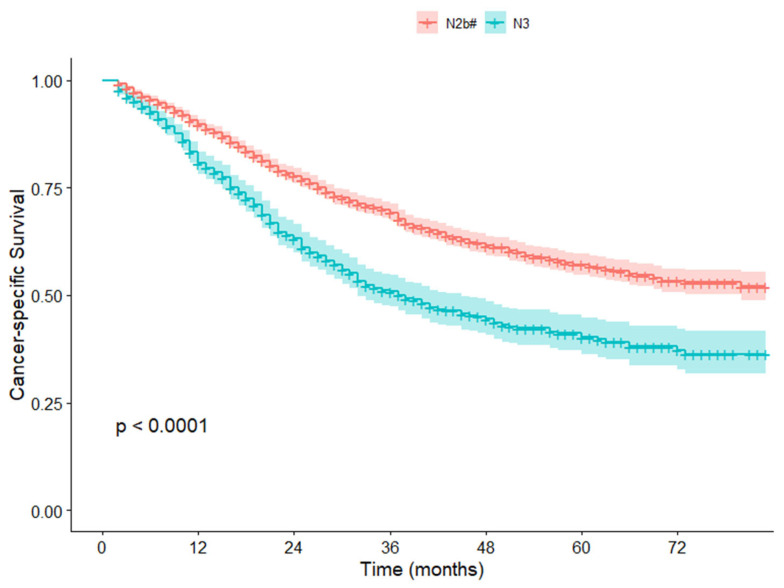
Cancer-specific survival curves for TxN2b#M0 and TxN3M0 patients in the SEER cohort.

In the SEER cohort, patients with TxN2bM0 were similarly divided into two groups based on the same cutoff value (stage N2b#: 2626 cases; stage N3: 894 cases). Survival analysis revealed that the 5-year CSS rate of stage N2b# patients was significantly better than that of stage N3 patients (5-year CSS rate: 57.1% vs. 40.2%, *p* < 0.001) (Figure 3).

### 3.5. Cox Analysis for CSS and Comparison of Survival Differences Between N2b Substage and Other N Groups in the SEER Cohort

Cox univariate and multivariate analyses were conducted to estimate the relationships between clinicopathological characteristics and CSS. Univariate analysis identified several important prognostic factors for CSS, including age, race, marital status, preoperative CEA, perineural invasion, differentiation, pT status, modified pN status, number of examined LNs, adjuvant radiotherapy, and adjuvant chemotherapy (all *p* < 0.001). Multivariate analysis confirmed that age, race, marital status, preoperative CEA, perineural invasion, differentiation, pT status, modified pN status, number of examined LNs, adjuvant radiotherapy, and adjuvant chemotherapy were significantly associated with CSS (all *p* < 0.05) (Table 2).

**Figure 4:**
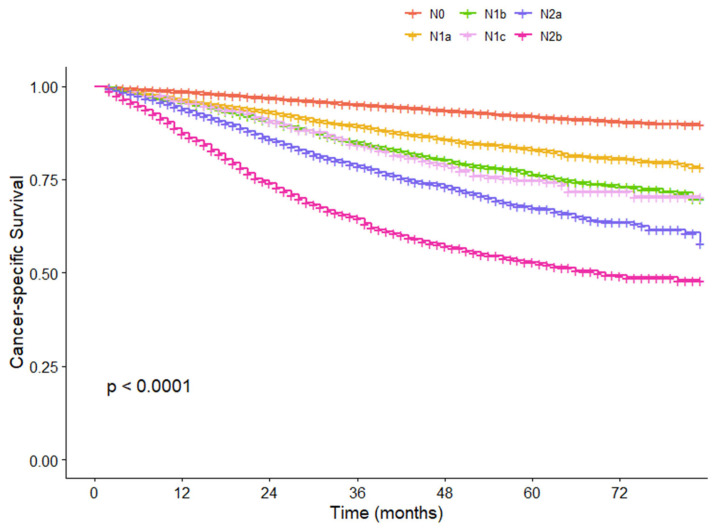
Cancer-specific survival curves for each N group before the N2b substage in the SEER cohort.

**Figure 5:**
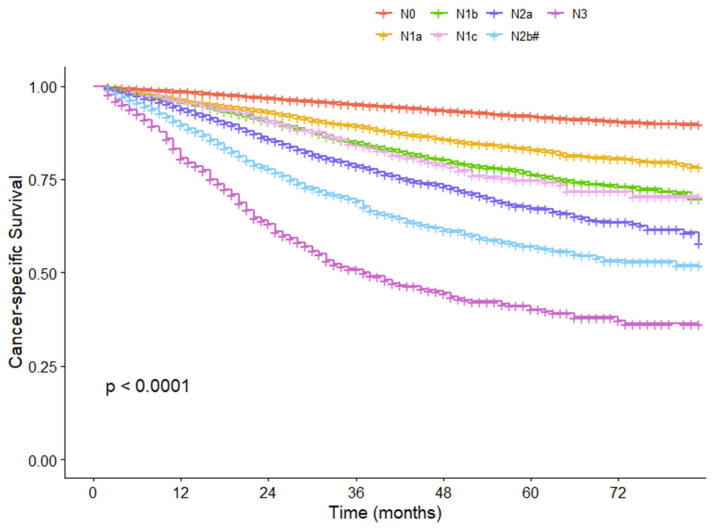
Cancer-specific survival curves for each N group after the N2b substage in the SEER cohort.

We further compared survival among different N groups before and after the introduction of the N2b substage. Before the introduction of the N2b substage, the 5-year CSS rate for N2b patients was 52.8%, while it was 92.0%, 83.1%, 76.3%, 74.8%, and 67.4% for those with N0, N1a, N1b, N1c, and N2a, respectively (*p* < 0.001) (Figure 4). Following the implementation of the N2b substage, the 5-year CSS rates of N2b# and N3 patients were 57.1% and 40.2%, respectively (*p* < 0.001) (Figure 5). The patients in stage N3 demonstrated a notably poorer prognosis in comparison to patients in other stage N categories. This finding provides a refined method for the prognostic stratification of patients with stage N2b disease.

### 3.6. Survival Difference Between TxN3M0 and TxN0-2b#M1 in the SEER Cohort

**Figure 6:**
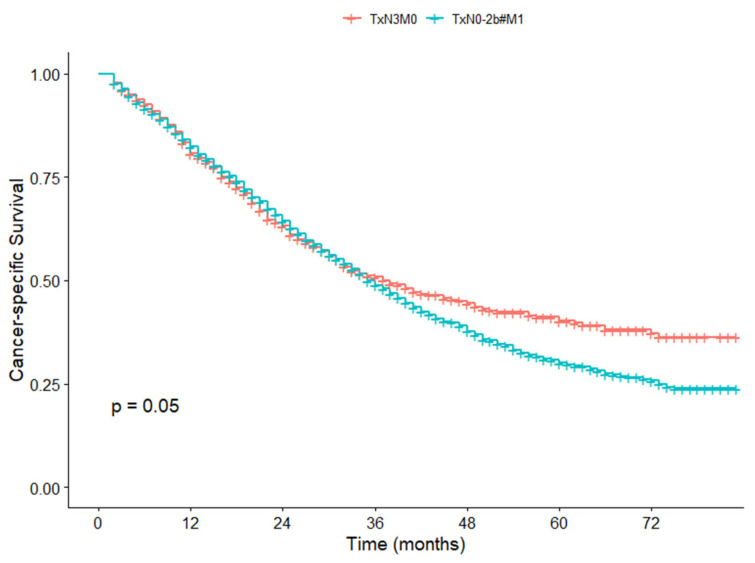
Cancer-specific survival curves for TxN3M0 and TxN0-2b#M1 patients in the SEER cohort.

**Figure 7:**
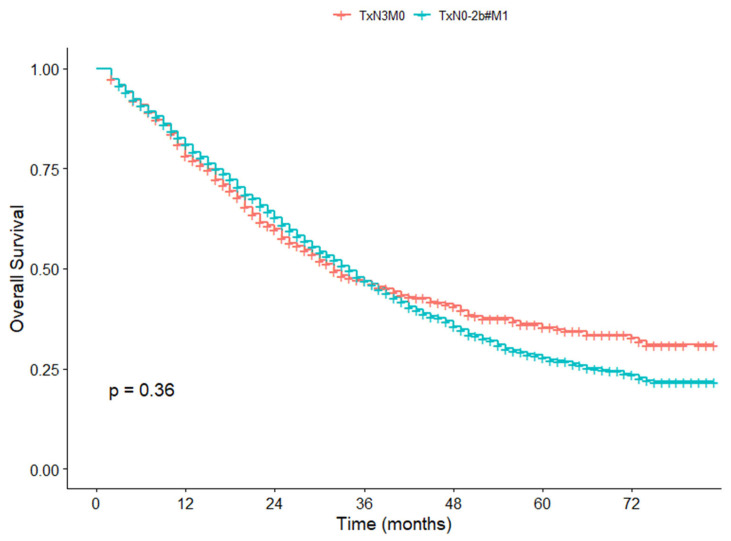
Overall survival curves for TxN3M0 and TxN0-2b#M1 patients in the SEER cohort.

To further underscore the adverse prognosis of N3, we conducted a comparative analysis of survival outcomes between two specific groups: TxN3M0 and TxN0-2b#M1, involving a total of 3573 patients. Among these, 894 patients had stage TxN3M0, and 2679 had TxN0-2b#M1 (We excluded 227 patients with TxN3M1 disease from the analysis). The results indicated that there was no significant difference in 5-year CSS rate between TxN3M0 and TxN0-2b#M1 patients (40.2% vs. 30.1%, *p* = 0.050) (Figure 6). In particular, to further validate our results, we compared the 5-year OS rates between these two groups of patients and found that they had no significant difference in survival rates (35.3% vs. 27.8%, *p* = 0.358) (Figure 7).

## 4. Discussion

The AJCC guidelines currently classify CRC patients with ≥7 LN metastases as stage N2b. However, there was substantial heterogeneity among the prognoses of stage N2b patients. That means the current TNM staging system fails to provide adequate prognostic information for this subgroup, so it is necessary to modify the N2b stage classification.

LN metastasis is a well-established independent prognostic factor in various solid tumors, such as pancreatic cancer, lung cancer, and colon cancer [12,13]. Previous studies have consistently shown that prognosis worsens as the number of positive LNs increases. For instance, Gunderson et al. reviewed the data from 35,829 rectal cancer patients in the SEER database and found that the patients with just one metastatic LN had a 3–10% higher 5-year survival rate compared to those with 2–3 LNs involved. Similarly, patients with 4 to 6 metastatic LNs had a 5–20% higher 5-year survival rate than those with ≥7 LNs affected [11]. Compton et al. emphasized that the regional LN metastasis significantly impacted the prognosis of CRC patients [5]. Therefore, the N2b stage alone may be insufficient to capture the prognostic diversity among these patients. It is not reasonable that all the patients with ≥7 metastatic LNs were classified into the same N stage.

In this study, we used X-tile software to determine an optimal cutoff value of 13 positive lymph nodes for stratifying patients with stage N2b disease, based on data from the NCC cohort. Using the new cutoff value, we subcategorized N2b patients into stage N2b# and N3. In the NCC cohort, we observed significant differences in survival between stage N2b# and N3 patients. These findings were validated in the SEER cohort. That means the new cutoff value can effectively stratify the prognoses of N2b patients across different cohorts. Moreover, multivariate analysis confirmed that the modified pN stage was an independent prognostic factor for survival. These results strongly underscore the necessity and significance of subcategorizing the current N2b stage.

Additionally, our analysis revealed that the N3 patients had the poorest prognosis among M0 patients and that survival was as poor as that of M1 patients. This observation suggests that when the number of regional metastatic LNs becomes sufficiently high (more than 13 positive LNs), the prognosis may resemble that of patients with distant metastasis. PLN in colorectal cancer (CRC) is not merely a staging parameter but a robust indicator of underlying aggressive tumor biology. Crucially, a high positive lymph node count directly correlates with key biological phenomena: Firstly, a high positive lymph node burden strongly reflects activation of the Epithelial–Mesenchymal Transition (EMT) program, which enables tumor cell invasion and metastasis. Tumors with high positive lymph node counts show significantly reduced E-cadherin (CDH1) expression [14]. E-cadherin downregulation disrupts cell–cell adhesion, facilitating detachment and lymphatic invasion. Elevated expression of VIM, CDH2, SNAIL, SLUG, TWIST, and ZEB1 characterizes primary CRC tumors with extensive lymph node metastasis [15]. Secondly, the CRC Consensus Molecular Subtype (CMS) classification provides a direct biological framework explaining high positive lymph node counts: CMS4 tumors are defined by prominent EMT activation, stromal invasion, TGF-β signaling, angiogenesis, and immune suppression [7]. This subtype demonstrates the highest frequency of lymph node metastases (N2 status) and distant spread compared to CMS1–3 [7].

Patients with positive LNs can benefit from adjuvant chemotherapy [16], and the adjuvant chemotherapy usually lasts 6 months. The results of the IDEA study suggested that 3 months of adjuvant chemotherapy was adequate for low-risk stage III patients (T1–3N1); however, high-risk stage III patients (T4 or N2) must receive adjuvant chemotherapy for 6 months in principle [17]. To a certain extent, the findings of the IDEA study changed the traditional concept of clinical practice. These findings indicated that using the same adjuvant chemotherapy scheme for all stage III colon cancer patients was inappropriate. The treatment scheme should be made based on the precise prognostic stratification. We should individually select the appropriate “patients” to receive the proper “adjuvant chemotherapy scheme” and “adjuvant chemotherapy cycle”. Precise staging and prognostic stratification allow for tailored treatment strategies, which can prevent both undertreatment and overtreatment to deliver optimal patient care. For stage N3 patients, future research should design large-scale randomized controlled clinical trials to further clarify their prognosis and provide evidence for stratified management. Our findings provide clues for future studies.

Currently, all N2b CRC patients are treated as stage III and treated with the same adjuvant therapy [4,18]. However, according to the results of our research, N2b CRC patients should be individually treated based on the prognostic stratification. Postoperative adjuvant treatment should be tailored according to patient prognosis and risk stratification, particularly for stage N3 cases, to align with precision medicine principles. Furthermore, enhanced follow-up is recommended to enable timely assessment of disease progression.

In this study, we first determined the optimal PLN cutoff value based on the NCC cohort to subdivide the N2b category. Subsequently, we verified the reliability of this classification using the SEER database. We found that the prognosis of stage N2b and N3 patients differed significantly in both the NCC and SEER cohorts. In N3 patients, whose prognosis is similar to M1, intensive treatment should be required.

This study has several limitations. Firstly, it is a retrospective study, which may introduce selection bias. Secondly, the SEER database has incomplete data; the chemotherapy regimen (e.g., FOLFOX vs. CAPOX), radiotherapy details (dose/fields), and molecular markers (e.g., MSI status, RAS/BRAF) are unavailable. These omissions weaken the multivariate Cox model’s ability to isolate PLN’s prognostic impact. Thirdly, survival differences between the N2b substage and other N stages in the NCC cohort have not been demonstrated due to limited samples. Prospective, multi-center, large-sample studies are expected to be carried out in the future to verify the conclusions of this study.

## 5. Conclusions

The current N2b stage is heterogeneous and should be subdivided into N2b# and N3 groups based on the number of positive LNs. The prognosis of the N3 patients with ≥13 positive LNs is significantly worse than that of the N2b# patients with 7–12 positive LNs. The N3 patients should receive individualized management and close follow-up.

## Figures and Tables

**Table 1:** Cox univariate and multivariate analyses for OS of 240 patients in the NCC cohort.

Characteristics		Univariate Analysis		Multivariate Analysis	
	No.	HR (95% CI)	*p*	HR (95% CI)	*p*
Gender			0.284		
Men	141	1.000			
Women	99	0.807 (0.545–1.194)			
Age (year)			<0.001		0.001
≤60	147	1.000		1.000	
>60	93	2.008 (1.373–2.936)		1.949 (1.300–2.923)	
Preoperative CEA			0.031		
Positive	67	1.000			
Negative	123	0.605 (0.389–0.941)	0.026		
Unknown	50	1.035 (0.628–1.705)	0.892		
Preoperative CA199			0.043		0.007
Positive	29	1.000		1.000	
Negative	159	0.550 (0.318–0.953)	0.033	0.382 (0.215–0.676)	0.001
Unknown	52	0.830 (0.449–1.536)	0.554	0.528 (0.276–1.010)	0.054
Surgical procedure			0.891		
Open	87	1.000			
Laparoscope	153	1.028 (0.694–1.522)			
Tumor location			0.009		0.004
Rectum	151	1.000		1.000	
Colon	89	0.561 (0.364–0.864)		0.512 (0.324–0.811)	
Vascular invasion			0.011		
Positive	97	1.000			
Negative	143	0.611 (0.417–0.893)			
Perineural invasion			0.025		0.007
Positive	77	1.000		1.000	
Negative	163	0.639 (0.432–0.946)		0.569 (0.378–0.858)	
Differentiation grade			0.013		
Well/Unknown	3	1.000			
Moderate	152	0.441 (0.107–1.808)	0.255		
Poor	85	0.769 (0.186–3.175)	0.717		
pT			0.004		0.002
T1–2	9	1.000		1.000	
T3	147	0.813 (0.294–2.243)	0.689	0.833 (0.289–2.404)	0.736
T4	84	1.576 (0.568–4.379)	0.382	1.774 (0.608–5.182)	0.294
pN			0.001		0.002
N2b#	163	1.000		1.000	
N3	77	1.966 (1.335–2.895)		1.869 (1.253–2.787)	
Number of LNs examined < 12			0.550		
Yes	6	1.000			
No	234	0.736 (0.270–2.010)			
Adjuvant radiation			0.970		
Yes	84	1.000			
No	156	1.008 (0.676–1.501)			
Adjuvant chemotherapy			<0.001		<0.001
Yes	206	1.000		1.000	
No	34	3.459 (2.199–5.439)		3.004 (1.842–4.901)	

OS: overall survival; LN: lymph node; HR: hazard ratio; CI: confidence interval; CEA: carcinoembryonic antigen.

**Table 2:** Cox univariate and multivariate analyses for CSS of 65,189 patients in the SEER cohort.

Characteristics		Univariate Analysis		Multivariate Analysis	
	No.	HR (95% CI)	*p*	HR (95% CI)	*p*
Gender			0.947		
Men	33,588	1.000			
Women	31,601	0.998 (0.953–1.046)			
Age (year)			<0.001		<0.001
≤60	25,207	1.000		1.000	
>60	39,982	1.598 (1.518–1.681)		1.697 (1.609–1.791)	
Race			<0.001		<0.001
White	50,283	1.000		1.000	
Black	7971	1.331 (1.247–1.420)	<0.001	1.249 (1.169–1.336)	<0.001
Other/Unknown	6935	0.890 (0.821–0.966)	0.005	0.812 (0.748–0.881)	<0.001
Marital status			<0.001		<0.001
Single	10,526	1.000		1.000	
Married	36,577	0.652 (0.613–0.695)	<0.001	0.741 (0.694–0.790)	<0.001
Widowed/divorced	13,756	1.044 (0.974–1.119)	0.222	0.983 (0.915–1.056)	0.633
Others	4330	0.750 (0.673–0.834)	<0.001	0.822 (0.738–0.915)	<0.001
Preoperative CEA			<0.001		<0.001
Positive	12,305	1.000		1.000	
Negative	25,021	0.408 (0.384–0.434)	<0.001	0.634 (0.596–0.675)	<0.001
Unknown	27,863	0.570 (0.539–0.602)	<0.001	0.825 (0.779–0.873)	<0.001
Tumor location			0.073		
Rectum	11,462	1.000			
Colon	53,763	1.058 (0.995–1.126)			
Perineural invasion			<0.001		<0.001
Positive	5612	1.000		1.000	
Negative	52,794	0.336 (0.317–0.357)	<0.001	0.729 (0.684–0.777)	<0.001
Unknown	6783	0.438 (0.402–0.477)	<0.001	0.808 (0.739–0.883)	<0.001
Differentiation grade			<0.001		<0.001
Well	6033	1.000		1.000	
Moderate	47,135	1.683 (1.511–1.874)	<0.001	1.238 (1.110–1.380)	<0.001
Poor/undifferentiation	10,045	3.693 (3.299–4.133)	<0.001	1.713 (1.527–1.923)	<0.001
Unknown	1976	1.076 (0.882–1.314)	0.470	1.259 (1.030–1.539)	0.024
pT			<0.001		<0.001
T1	11,886	1.000		1.000	
T2	11,467	1.991 (1.706–2.323)	<0.001	1.801 (1.541–2.105)	<0.001
T3	33,754	6.244 (5.490–7.102)	<0.001	4.208 (3.681–4.810)	<0.001
T4	8082	17.679 (15.501–20.164)	<0.001	9.455 (8.228–10.865)	<0.001
pN			<0.001		<0.001
N0	41,004	1.000		1.000	
N1a	7666	2.240 (2.077–2.416)	<0.001	1.992 (1.840–2.158)	<0.001
N1b	7465	3.165 (2.956–3.388)	<0.001	2.642 (2.454–2.844)	<0.001
N1c	1017	3.301 (2.814–3.873)	<0.001	2.298 (1.955–2.702)	<0.001
N2a	4517	4.630 (4.306–4.977)	<0.001	3.517 (3.249–3.809)	<0.001
N2b#	2626	6.976 (6.451–7.543)	<0.001	4.944 (4.534–5.392)	<0.001
N3	894	12.237 (11.032–13.575)	<0.001	8.170 (7.298–9.146)	<0.001
Number of examined LNs < 12			<0.001		<0.001
Yes	9212	1.000		1.000	
No	55,977	0.851 (0.799–0.905)		0.570 (0.535–0.608)	
Adjuvant radiation			<0.001		0.004
Yes	2824	1.000		1.000	
No	62,365	0.700 (0.637–0.770)		0.866 (0.786–0.955)	
Chemotherapy			<0.001		<0.001
Yes	21,671	1.000		1.000	
No	43,518	0.574 (0.547–0.601)		1.531 (1.448–1.619)	

CSS: cancer-specific survival; HR: hazard ratio; CI: confidence interval; CEA: carcinoembryonic antigen.

## Data Availability

We may balance the potential benefits and risks for each request and then provide the data that could be shared. Data are available from the corresponding author upon reasonable request. The SEER data can be obtained from https://seer.cancer.gov/.
